# The Management of Navicular Dislocation and Multifocal Fractures Following a Fall

**DOI:** 10.7759/cureus.64581

**Published:** 2024-07-15

**Authors:** Rawaa J Khan, Abdulrahman J Khan, Haneen A Alhelali, Anas S Almoabbdi, Bushra Alshanqiti

**Affiliations:** 1 Medicine, College of Medicine, Umm Al-Qura University, Jeddah, SAU; 2 Radiology, King Abdulaziz University Faculty of Medicine, Makkah, SAU; 3 Orthopedic Surgery, King Fahad General Hospital, Jeddah, SAU; 4 Orthopedic Surgery, Armed Forces Hospital, Makkah, SAU; 5 Orthopedic Surgery, King Abdullah Medical Complex, Jeddah, SAU

**Keywords:** management of navicular dislocation, navicular bone, midfoot injury, multifocal fractures, isolated navicular dislocation

## Abstract

Midfoot injuries, encompassing navicular dislocations and fractures of the navicular bone, present unique challenges in trauma care. We report the case of a 27-year-old male who suffered a fall down a flight of stairs, resulting in navicular dislocation and multifocal fractures of the midfoot. The patient underwent a comprehensive management plan, including admission, closed reduction, and internal fixation with parallel K-wires. Radiological evaluations confirmed the extent of the injuries. Postoperatively, the patient received tailored care, incorporating pain management, antibiotics, and thromboprophylaxis. The successful outcome underscores the importance of a structured approach in addressing midfoot injuries, contributing to the existing literature on optimal management strategies. This case report serves as a valuable addition to the growing body of knowledge guiding clinicians in the effective treatment of navicular dislocations and navicular fractures.

## Introduction

Traumatic injuries resulting from falls are frequent occurrences in emergency departments worldwide, posing a significant burden on healthcare systems [1]. Such incidents can lead to a diverse array of injuries, ranging from mild contusions to severe fractures and dislocations [1,2]. Among the myriad consequences of falls, navicular dislocations and fractures affecting the midfoot, particularly the navicular bone, present unique challenges in terms of diagnosis, treatment, and postoperative care [3,4].

The midfoot, a complex anatomical region comprising the navicular bone, cuboid bone, cuneiforms, and metatarsals, plays a crucial role in weight-bearing and ambulation [5]. The navicular bone, situated on the dorsal aspect of the midfoot, is particularly susceptible to fractures due to its location and the forces exerted during falls [5,6]. Dislocations involving the navicular structures in this region compound the complexity of these injuries, necessitating prompt and comprehensive management to achieve optimal outcomes [3].

The clinical presentation of midfoot injuries can vary, making accurate diagnosis pivotal for appropriate treatment planning [7]. Patients often present with localized pain, swelling, and limited range of motion, requiring a meticulous examination and a thorough radiological assessment [8]. Imaging modalities such as X-rays, computed tomography (CT), and magnetic imaging resonance (MRI) scans play a crucial role in delineating the extent of fractures, dislocations, and associated soft tissue injuries [9]. Understanding the specific patterns of midfoot fractures, such as navicular fractures, is crucial for tailoring treatment strategies and achieving optimal functional outcomes [7].

Orthopedic intervention is frequently indicated in cases involving navicular dislocations and multifocal fractures of the midfoot [10]. Open reduction and internal fixation (ORIF) is commonly employed to restore anatomical alignment, stability, and function [11]. The choice of fixation devices, such as K-wires or plates with screws, depends on the specific characteristics of the fractures and the surgeon's preference [12]. Successful management extends beyond the operating room, encompassing meticulous preoperative planning, intraoperative decision-making, and comprehensive postoperative care [13,14].

Postoperative care comprises integral components such as pain management, wound care, and monitoring for complications such as infection and thrombosis [15]. Rehabilitation strategies, including weight-bearing protocols and physiotherapy, are tailored to each patient's specific injuries and overall health status [15,16]. Additionally, regular follow-up appointments and imaging studies are essential to track the progress of fracture healing, assess the stability of the fixation devices, and address any emerging concerns [16].

This manuscript presents a comprehensive case report detailing the evaluation, surgical intervention, and postoperative care of a patient presenting with navicular dislocation and multifocal fractures of the midfoot following a fall. The case serves as a paradigm for understanding the complexities of midfoot injuries and highlights the importance of a multidisciplinary approach in achieving successful outcomes.

## Case presentation

A 27-year-old male presented to the emergency department following a traumatic fall down a flight of stairs. The patient reported slipping while descending one floor, resulting in a significant impact on the lower extremities. Initial evaluation, as per the primary survey, revealed stable vital signs with a Glasgow Coma Scale score of 15/15. The airway was intact, and the patient wore a cervical collar for precautionary measures. Breath sounds were clear, and oxygen saturation on room air was 99%. Abdominal examination showed softness, and the pelvis was stable. Notably, the secondary survey identified left ankle swelling and tenderness at the lateral malleolus and signs of a right tibial fracture.

Radiological investigations, including X-rays, were promptly ordered to assess the extent of the injuries. The imaging revealed a navicular dislocation along with a cuboidal bone fracture in the right foot. Further imaging indicated a comminuted fracture of the navicular bone with dorsal dislocation of bone fragments. Additionally, there were fractures involving the second and third metatarsal bases and likely fractures of the intermediate and lateral cuneiform bones. These findings were confirmed through a detailed CT scan, which also highlighted marked soft tissue edema around the affected area.

**Figure 1:**
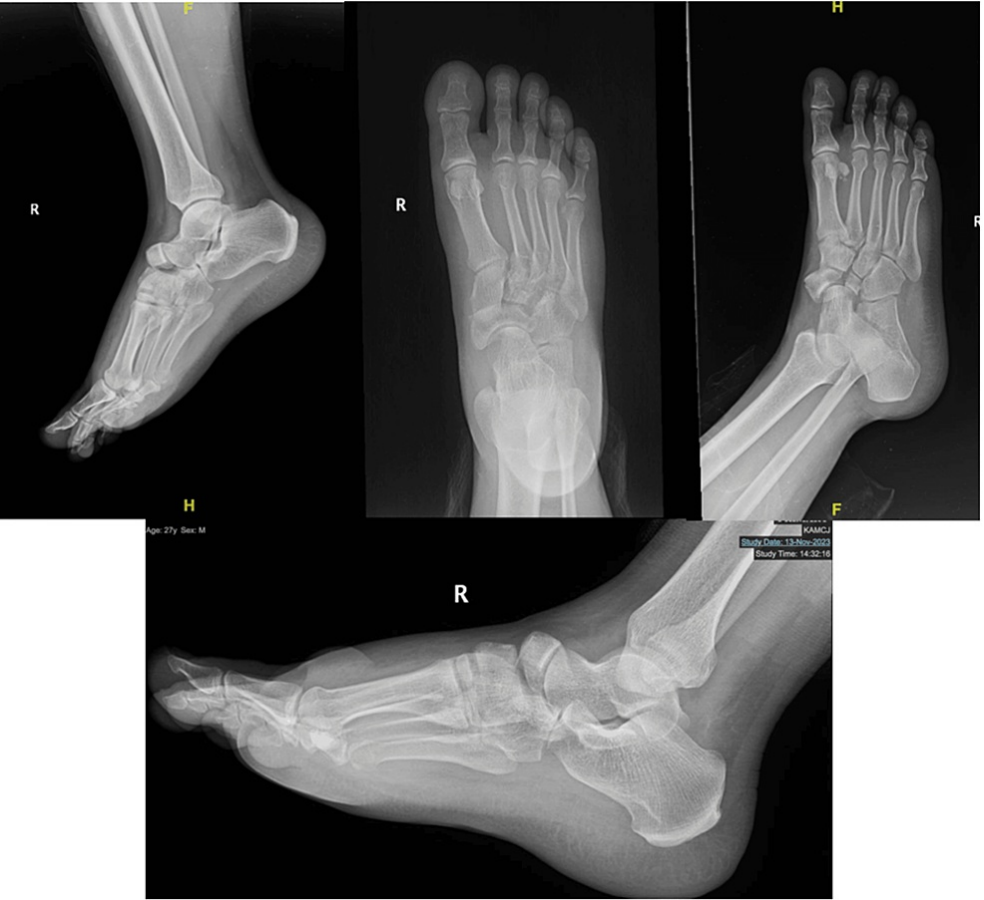
Preoperative X-ray films.

Upon consultation with the orthopedic team, the patient underwent a thorough examination. Clinical findings included tenderness, swelling, and limited range of motion in the right foot. Dorsalis pedis pulse was non-palpable, and X-rays confirmed navicular dislocation and associated fractures (Figure 1). A definitive plan for management was established, comprising admission, closed reduction, post-reduction X-rays, pain management, the application of a backslab, leg elevation, and anti-inflammatory measures.

Preoperative planning involved obtaining informed consent from the patient for the open reduction and internal fixation (ORIF) of the navicular bone. The choice between using K-wires and a plate and screws intraoperatively based on the surgeon's judgment was necessary. The patient was scheduled for surgery the following day, with nil per os (NPO) instructions starting at midnight. Intravenous (IV) fluids were administered, and prophylactic antibiotics (cefazolin) were initiated.

**Figure 2:**
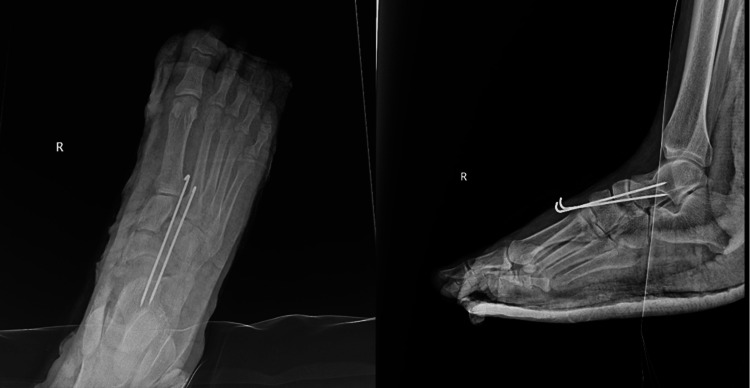
Postoperative X-ray films showing the fixation.

In the operating room, the patient underwent general anesthesia and was positioned supine. The fracture site was identified using fluoroscopy, and a closed reduction of the dislocated navicular bone was performed. Fixation was achieved using two parallel K-wires (Figure 2). The confirmation of reduction and fixation was obtained through intraoperative fluoroscopy. Concurrently, bulla evacuation was carried out, and a dressing was applied. A below-knee backslab was then meticulously applied to maintain the corrected position.

Postoperatively, the patient was prescribed a carefully planned regimen for pain management and prophylaxis. This included intravenous paracetamol, pethidine, cefazolin, and omeprazole. Subcutaneous enoxaparin was initiated for thromboembolism prophylaxis. A strict nil per os (NPO) regimen was maintained until the patient achieved full recovery, after which oral feeding was gradually reintroduced. Additionally, postoperative orders included regular right foot X-rays to monitor the stability of the fixation and leg elevation on two pillows to manage postoperative edema.

The patient was closely monitored during the postoperative period for any signs of infection, thrombosis, or other complications. Rehabilitation strategies, including weight-bearing protocols and physiotherapy, were tailored to the specific injuries and the patient's overall health status. Follow-up appointments were scheduled to assess fracture healing progress and ensure the success of the surgical intervention.

**Figure 3:**
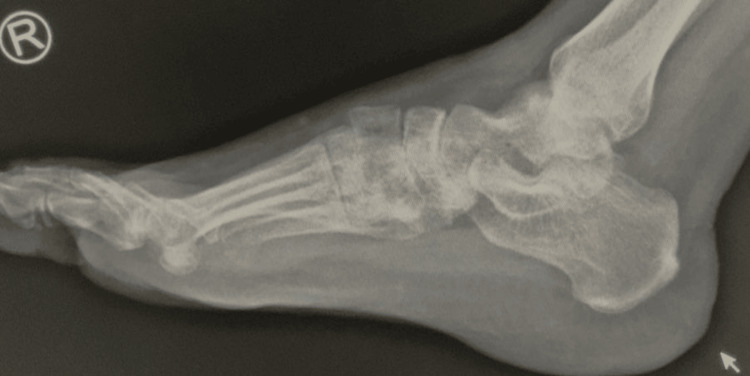
Six-month follow-up X-ray of the right foot.

At the six-month follow-up visit, the patient walked in completely weight-bearing, there was no obvious deformity, and the patient denied any pain. X-ray in lateral and AP views showing intermediate cuboid not clearly visualized could be due to severe osteopenia or subluxation (Figure 3).

This case report provides a detailed account of the evaluation, surgical intervention, and postoperative care of a patient with navicular dislocation and multifocal fractures of the midfoot following a traumatic fall. The chronological presentation of the case aims to offer insights into the complexities of managing such injuries and emphasizes the importance of a structured, multidisciplinary approach for optimal patient outcomes.

## Discussion

Midfoot injuries, particularly those involving navicular dislocations and fractures of the navicular bone, represent a challenging subset of traumatic orthopedic conditions [3,4]. The midfoot's intricate anatomy and biomechanical significance in weight-bearing make injuries to this region consequential and necessitate a nuanced approach to diagnosis and treatment [5]. Navicular dislocations, characterized by a disruption of the normal alignment of the bones along with vascular compromise, often demand urgent intervention to mitigate long-term complications [7]. Fractures of the navicular bone, although less common than other midfoot fractures, pose unique challenges due to their location and the potential for concurrent injuries [10].

In this case report, we present a 27-year-old male who sustained navicular dislocation and multifocal fractures of the midfoot following a fall down a flight of stairs. The patient's clinical presentation included left ankle swelling, tenderness at the lateral malleolus, and a suspected right tibial fracture. Radiological examinations, including X-rays and CT scans, revealed a navicular dislocation involving the cuboidal bone, a comminuted fracture of the navicular bone with dorsal dislocation of bone fragments, and associated fractures of the second and third metatarsal bases and intermediate and lateral cuneiform bones. The orthopedic team executed a comprehensive management plan involving admission, closed reduction, fixation with two parallel K-wires, post-reduction X-rays, pain management, backslab application, leg elevation, and anti-inflammatory measures.

The primary objective in the management of this case was the restoration of the anatomical alignment, stability, and function of the midfoot [7,9]. Closed reduction and internal fixation with parallel K-wires proved successful in achieving this goal [9,17]. The choice of K-wires as the fixation method was based on intraoperative considerations and the surgeon's judgment, highlighting the importance of adaptability in the face of complex fractures [17-19].

The postoperative course was marked by meticulous care to mitigate potential complications. Pain management, including intravenous paracetamol and pethidine, was administered judiciously, aligning with current recommendations for multimodal analgesia in orthopedic procedures [17]. Prophylactic measures, such as antibiotics and enoxaparin, were implemented to minimize the risk of infection and thromboembolism, respectively [15].

Comparisons with existing literature reveal a varied landscape in the management of navicular dislocations and navicular fractures [14-19]. While closed reduction with K-wires is a well-established approach, the choice of fixation method may vary among surgeons, with some opting for plate and screw constructs [17,18]. The decision often hinges on the specific fracture characteristics, surgeon preference, and institutional practices.

The successful outcomes observed in this case align with studies advocating for prompt intervention, anatomical reduction, and stable fixation in navicular dislocations and navicular fractures [4,19,20]. Postoperative care, including strict nil per os (NPO) adherence, leg elevation, and regular monitoring through X-rays, mirrors established protocols in the literature [17].

## Conclusions

This case report sheds light on the complexities of managing navicular dislocations and multifocal fractures of the midfoot following a traumatic fall. The successful outcome achieved through a structured and multidisciplinary approach underscores the importance of timely intervention, comprehensive preoperative planning, and meticulous postoperative care. The nuances presented in this case contribute to the growing body of literature guiding the management of midfoot injuries, emphasizing the need for individualized strategies tailored to the specific characteristics of each case.
